# Supplementary material for: Two cysteine dioxygenase homologs contribute to the fitness and virulence of a human pathogen
Source: Microbiol Spectr. 2026 Aug 19;14(10):e01414-26. doi: 10.1128/spectrum.01414-26 (PMC13689094; doi:10.1128/spectrum.01414-26)
Supplement: Supplementary file 1 [file spectrum.01414-26-s0001.pdf]

Two cysteine dioxygenase homologs contribute to the fitness and virulence of a human pathogen

Argha Saha[1], Denisa Petráčková[1], Jana Holubová[2], Martin Beles[1], Ivana Čurnová[1], Ondřej Staněk[2], and Branislav Večerek[1]*

Supplementary data

Supplementary Figures S1-S4

Supplementary Table S1

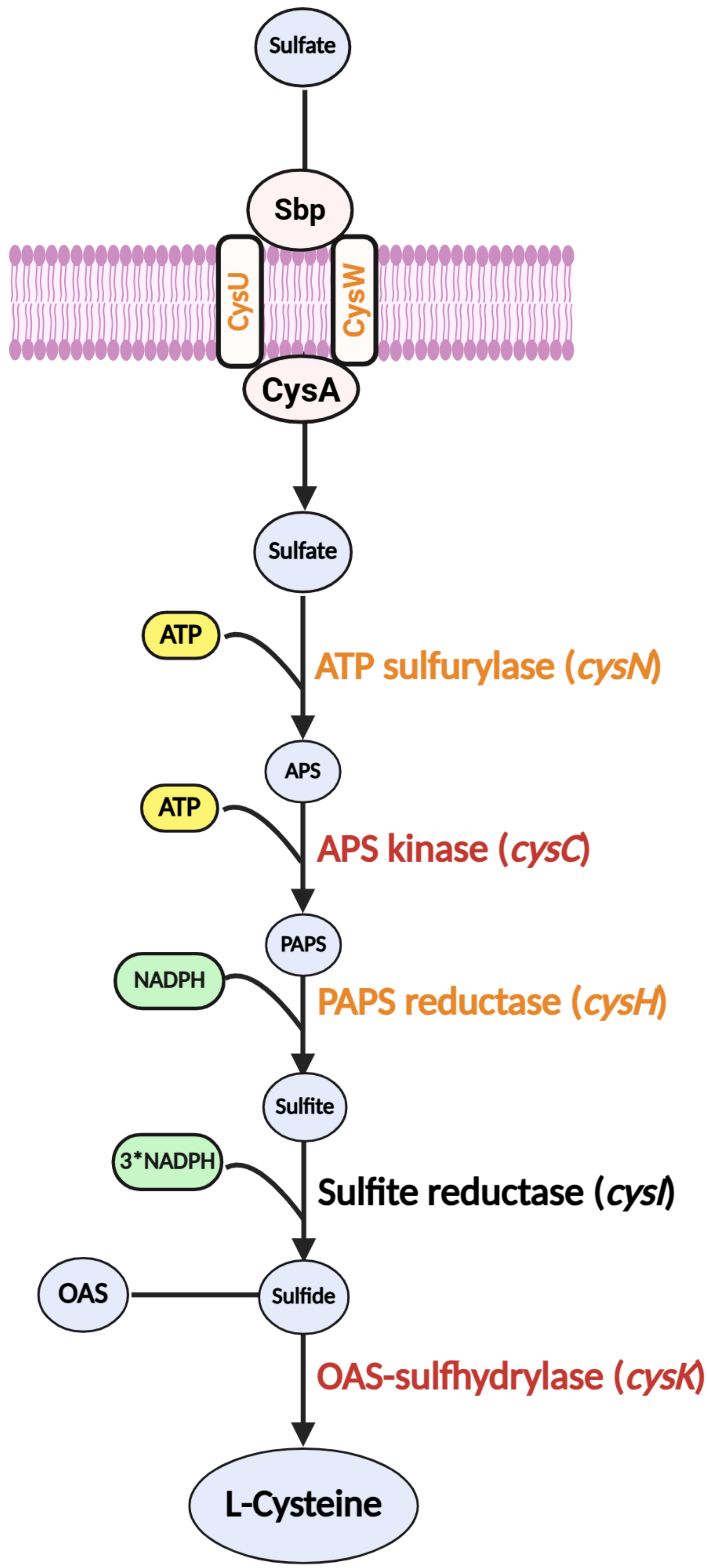

Fig. S1. *Bordetella pertussis* has decayed or lost several genes in the sulfate assimilation pathway. The schematic shows the sulfate assimilation pathway in bacteria. Genes that were decayed, such as *cysUW*, *cysN*, and *cysH*, or lost, such as *cysC* and *cysK*, in *B. pertussis* are shown in orange and red, respectively.

A

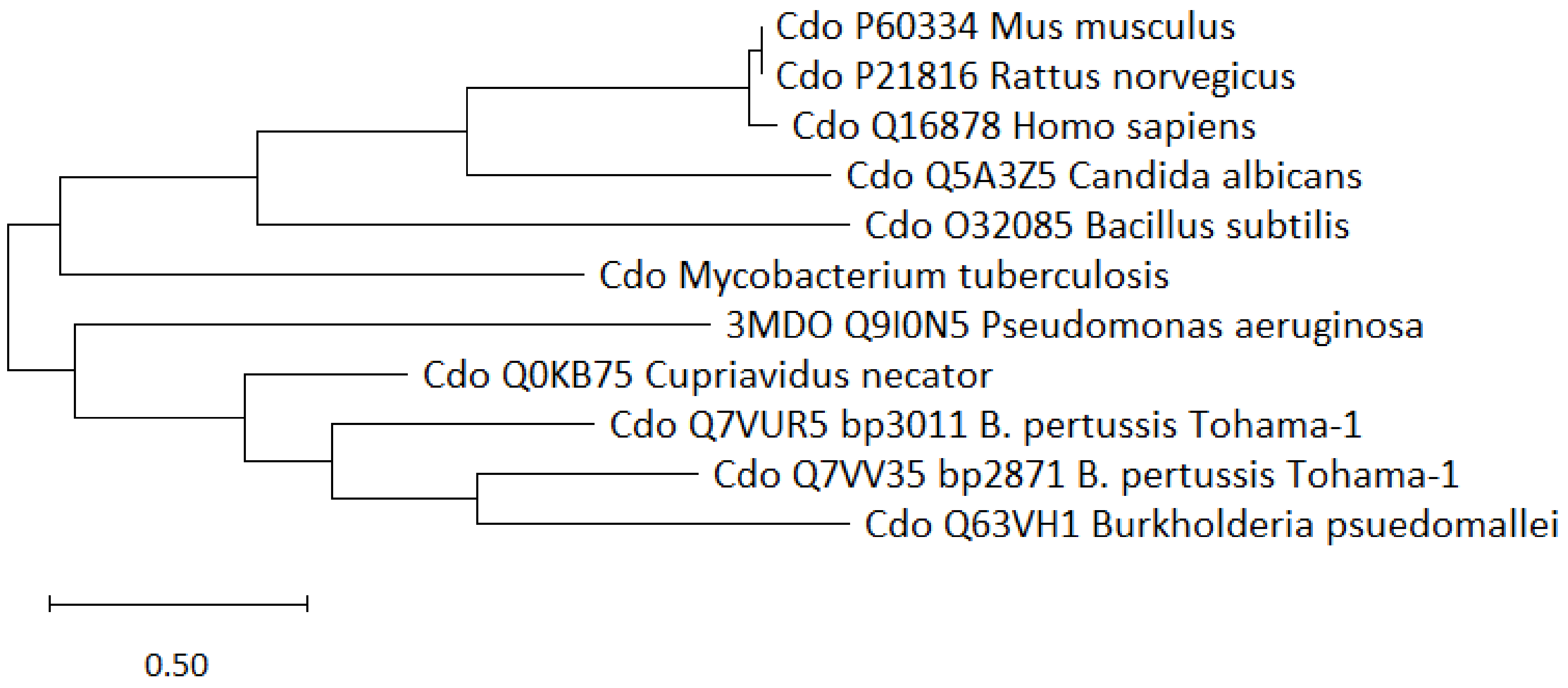

Cdo P60334 Mus musculus
Cdo P21816 Rattus norvegicus
Cdo Q16878 Homo sapiens
Cdo Q5A3Z5 Candida albicans
Cdo O32085 Bacillus subtilis
Cdo Mycobacterium tuberculosis
3MDO Q9I0N5 Pseudomonas aeruginosa
Cdo Q0KB75 Cupriavidus necator
Cdo Q7VUR5 bp3011 B. pertussis Tohama-1
Cdo Q7VV35 bp2871 B. pertussis Tohama-1
Cdo Q63VH1 Burkholderia psuedomallei
0.50

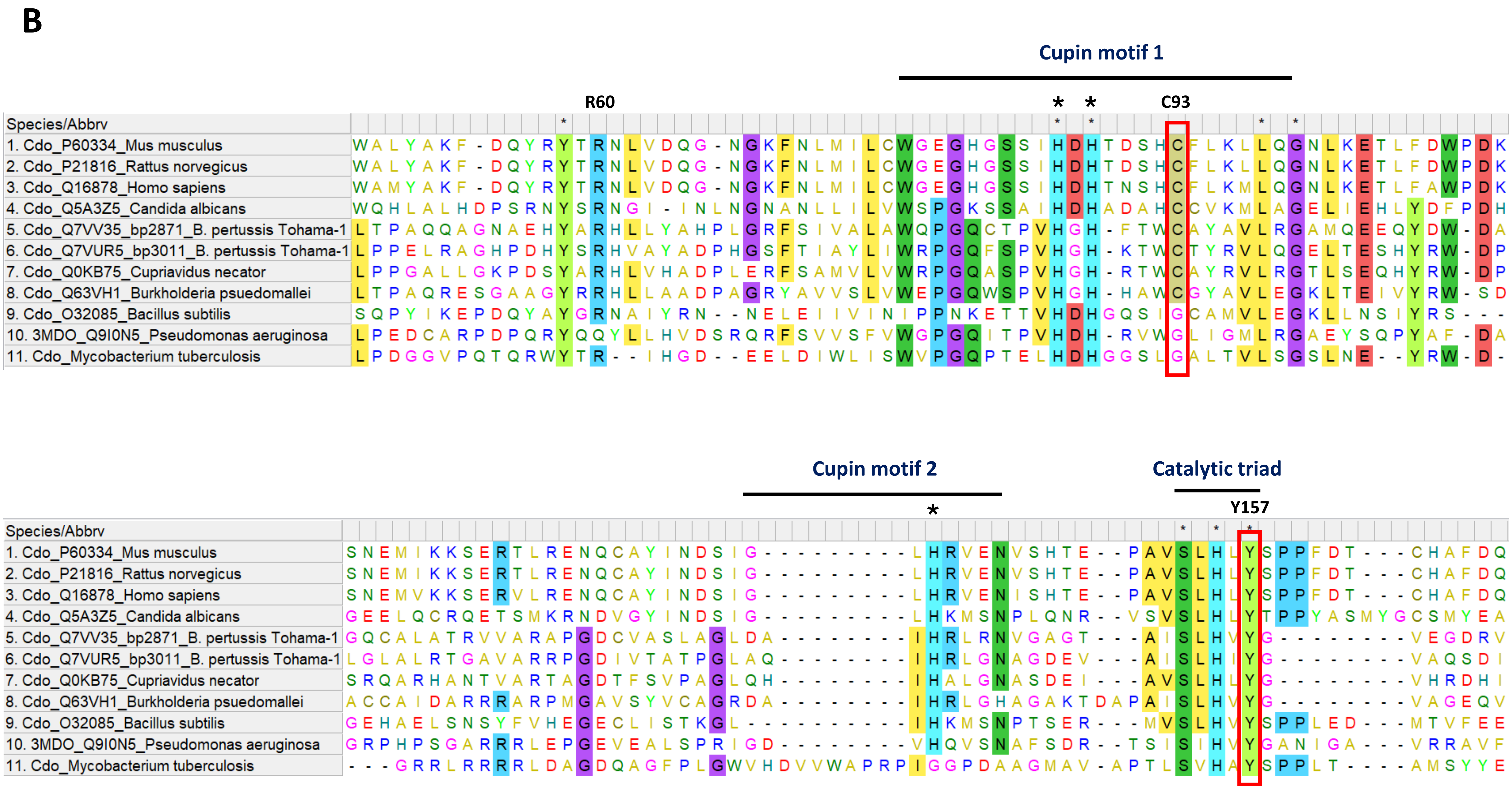
B
Cupin motif 1
R60
C93
Species/Abbrv
1. Cdo_P60334_Mus musculus
2. Cdo_P21816_Rattus norvegicus
3. Cdo_Q16878_Homo sapiens
4. Cdo_Q5A3Z5_Candida albicans
5. Cdo_Q7VV35_bp2871_B. pertussis Tohama-1
6. Cdo_Q7VUR5_bp3011_B. pertussis Tohama-1
7. Cdo_Q0KB75_Cupriavidus necator
8. Cdo_Q63VH1_Burkholderia psuedomallei
9. Cdo_O32085_Bacillus subtilis
10. 3MDO_Q9I0N5_Pseudomonas aeruginosa
11. Cdo_Mycobacterium tuberculosis
Cupin motif 2
Catalytic triad
Y157
Species/Abbrv
1. Cdo_P60334_Mus musculus
2. Cdo_P21816_Rattus norvegicus
3. Cdo_Q16878_Homo sapiens
4. Cdo_Q5A3Z5_Candida albicans
5. Cdo_Q7VV35_bp2871_B. pertussis Tohama-1
6. Cdo_Q7VUR5_bp3011_B. pertussis Tohama-1
7. Cdo_Q0KB75_Cupriavidus necator
8. Cdo_Q63VH1_Burkholderia psuedomallei
9. Cdo_O32085_Bacillus subtilis
10. 3MDO_Q9I0N5_Pseudomonas aeruginosa
11. Cdo_Mycobacterium tuberculosis

Fig. S2. Phylogenetic analysis and sequence alignment of *B. pertussis* CDO homologs to well-characterized thiol dioxygenases . (A) Phylogenetic tree inferred from sequences of selected thiol dioxygenases using the maximum likelihood method. The CDO homologs BP2871 (Q7VV35) and BP3011 (Q7VUR5) from *Bordetella pertussis* were compared with CDOs from *Mus musculus* (Uniprot ID P60334), *Rattus nervegicus* (P21816), *Homo sapiens* (Q16878), *Candida albicans* (Q5A3Z5), *Cupriavidus necator* (Q0KB75), *Burkholderia pseudomallei* (Q63VH1), *Mycobacterium tuberculosis*, and 3-mercaptopropionate dioxygenase (3MDO) from *Pseudomonas aeruginosa* (Q9ION5). The phylogeny was inferred using the maximum likelihood method and Jones-Taylor-Thornton model of amino acid substitutions, and the tree with the highest log likelihood is shown. The analytical procedure encompassed 11 amino acid sequences with 284 positions in the final dataset. Evolutionary analyses were conducted in MEGA 12. **(B)** Sequence alignment showing conserved residues within thiol dioxygenases used for phylogenetic tree analysis (panel A). Legend (amino acid residues are numbered according to *Mus musculus* CDO): R60, aids in cysteine recognition and distinguishes CDOs from 3MDOs; C93 and Y157 (red boxes) form a covalent crosslink in mammalian CDOs; *, histidine triad that coordinates the Fe(II) ion; regions corresponding to cupin motifs and catalytic triad are shown with solid bars.

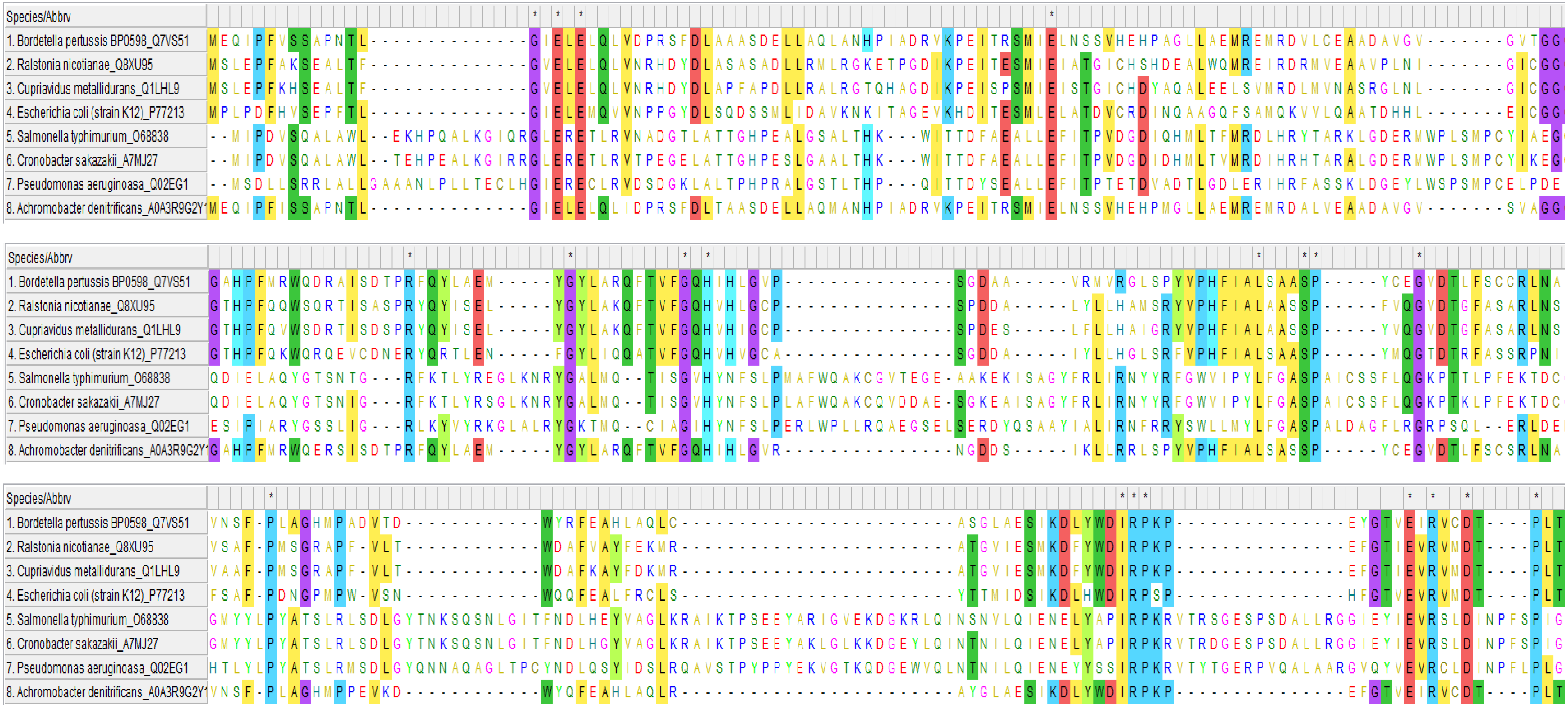
Species/Abbrv
1. Bordetella pertussis BP0598_Q7VS51
2. Ralstonia nicotianae_Q8XU95
3. Cupriavidus metallidurans_Q1LHL9
4. Escherichia coli (strain K12)_P77213
5. Salmonella typhimurium_O68838
6. Cronobacter sakazakii_A7MJ27
7. Pseudomonas aeruginosa_Q02EG1
8. Achromobacter denitrificans_A0A3R9G2Y1

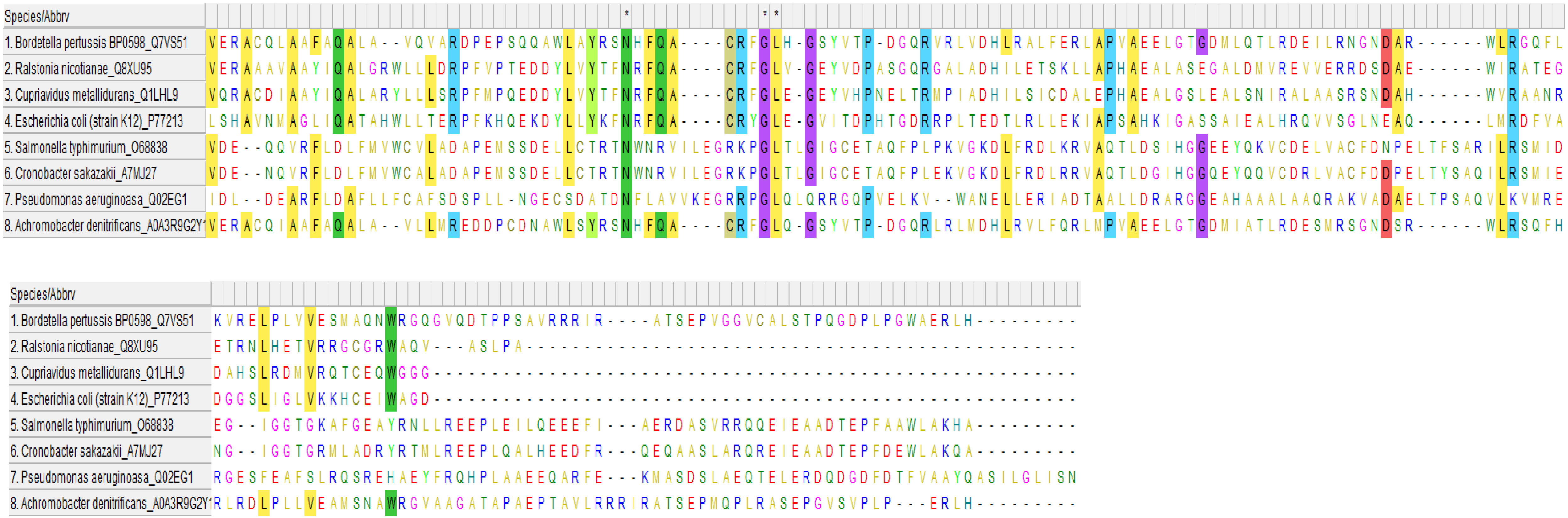

Fig. S3. The homology of the putative GshA protein BP0598 to other GshA proteins. (A) Sequence alignment of the putative glutamate-cysteine ligase (GshA) BP0598 (Uniprot ID Q7VS51) with other GshA proteins from *Ralstonia nicotinae* (Q8XU95), *Cupravidus metallidurans* (Q1LHL9), *Escherichia coli* (p77213), *Salmonella Typhimurium* (O68838), *Cronobacter sakazakii* (A7MJ27), *Pseudomonas aeruginosa*(Q8ZNK7), *Achromobacter denitrificans* (A0A3R9G2Y1). The analysis was conducted in MEGA 12.

A

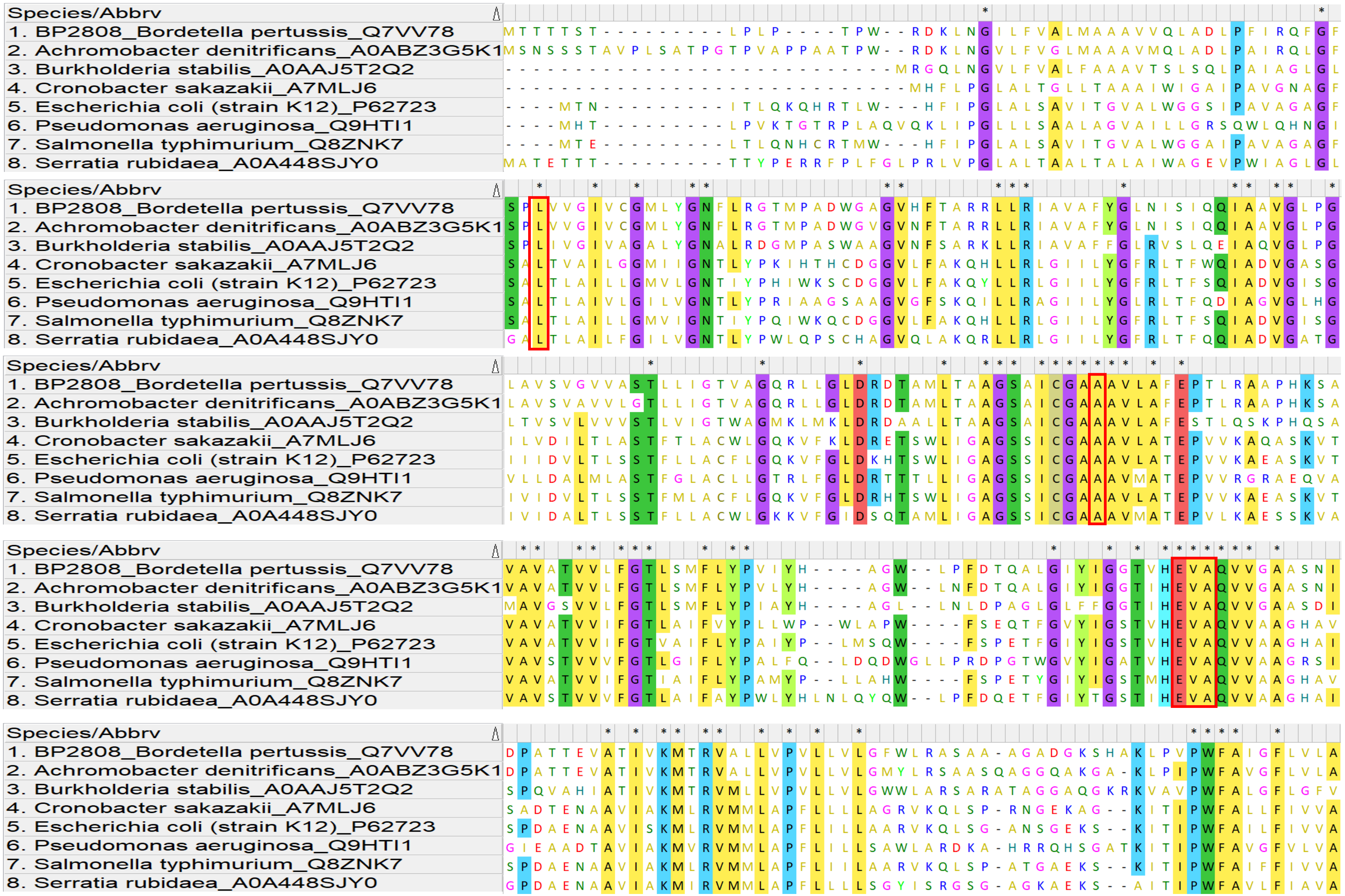
Species/Abbrv
1. BP2808_Bordetella pertussis_Q7VV78
2. Achromobacter denitrificans_A0ABZ3G5K1
3. Burkholderia stabilis_A0AAJ5T2Q2
4. Cronobacter sakazakii_A7MLJ6
5. Escherichia coli (strain K12)_P62723
6. Pseudomonas aeruginosa_Q9HTI1
7. Salmonella typhimurium_Q8ZNK7
8. Serratia rubidaea_A0A448SJY0

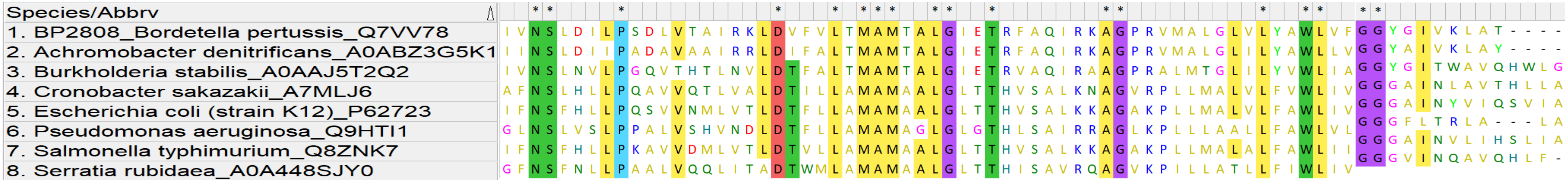

B

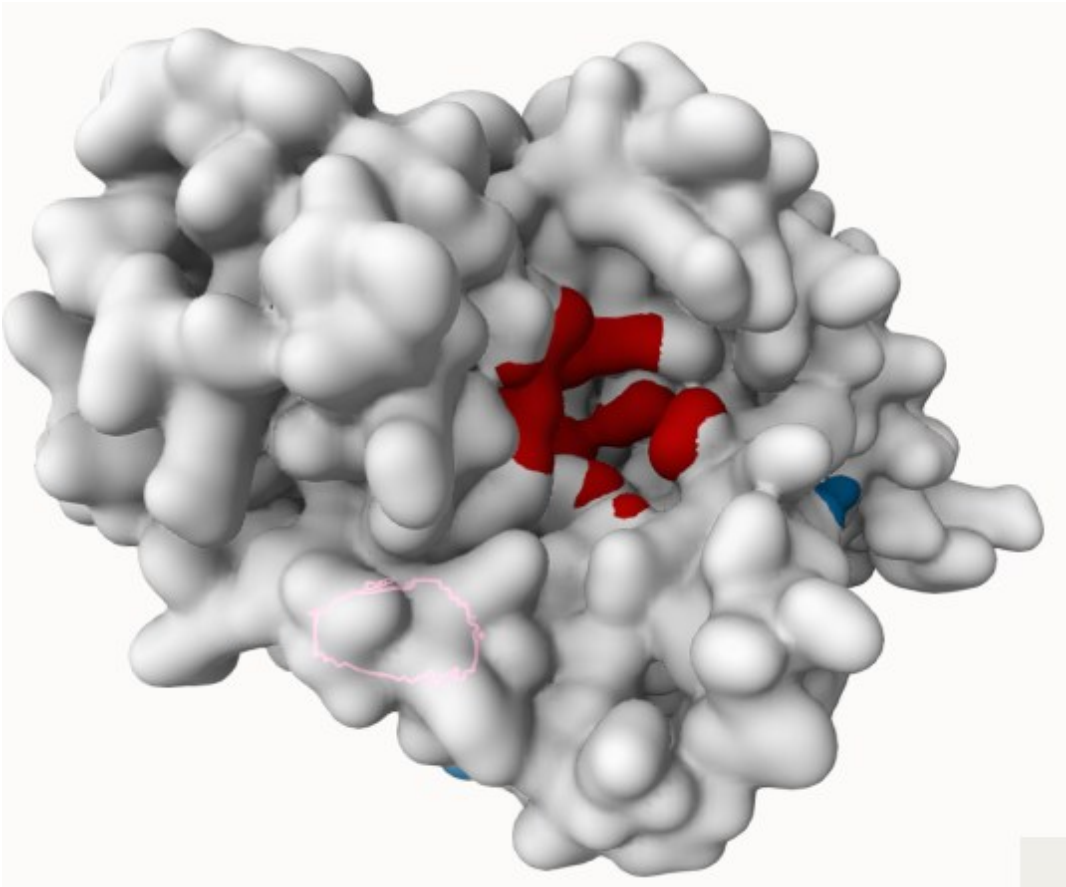

Fig. S4. The exporter BP2808 is homologous to the YeiH family of exporters. (**A**) Sequence alignment of the YeiH exporter BP2808 (Uniprot ID Q7VV78) with other YeiH family exporters from *Achromobacter denitricans* (A0ABZ3G5K1), *Burkholderia stabilis* (A0AAJ5T2Q2), *Cronobacter sakazakii* (A7MLJ6), *Escherichia coli* (P62723), *Pseudomonas aeruginosa* (Q9HTI1), *Salmonella Typhimurium* (Q8ZNK7), and *Serratia rubidaea* (A0A448SJY0). Amino acid residues (Leu48, Ala148, Glu208, Val209, Ala210) predicted to form the ligand binding site are shown with red boxes. The analysis was conducted in MEGA 12. (**B**) Localization of amino acid residues (in red) predicted to participate in the ligand binding site on the surface of BP2808 exporter. Visualization and prediction were performed using PrankWeb, a computational tool for predicting ligand binding sites (https://elixir-europe.org/services/prankweb).

TABLE S1. Primers used in this study

Purpose of the primers or probes	Sequence (5′-> 3′)*
Construction of the Δ*bp0598* mutant (upstream region)	F: ATA*ACTAGT*GACAAGATGGGCATCGACC<br>R: ATA*GCTAGC*TTCCATCTCCGACTCCTTG
Construction of the Δ*bp0598* mutant (downstream region)	F: ATA*GCTAGC*TTCCTCAAGGTGCGCGAAC<br>R: ATA*GAATTC*GTCGAGAACGGTTTTGCC
Construction of the Δ*bp2808* mutant (upstream region)	F: ATA*ACTAGT*GGAGGACTGCGTCATGCAAC<br>R: ATA*GCTAGC*TTCGGCGGCTACGGAATCG
Construction of the Δ*bp2808* mutant (downstream region)	F: ATA*GCTAGC*GGTCATAGCGCCGTCAGC<br>R: ATA*GAATTC*CGACGATCTGGCGCGTATTG
Construction of the *bp2871*$_{C119G}$ mutant (upstream region)	F: AGGGCGGCCGCACTAGTGAGACGAGATCAAGGGCCACAAGC<br>R: CATGGCGCCGCGCAGTACTGCGTAGGCGC**C**CCAGGTGAAATGG
Construction of the *bp2871*$_{C119G}$ mutant (downstream region)	F: CCATTTCACCTGG**G**GCGCCTACGCAGTACTGCGCGGCGCCATG<br>R: CGCGTGGATCCGAATTCGAGCCTATCGACCACGCCATGCT
Construction of the *bp3011*$_{C101G}$ mutant (upstream region)	F: AGGGCGGCCGCACTAGTGAGGGTCCCAGCATCATCGCGTC<br>R: CTGCAGTACTCGGTAGGTGC**C**CCAGGTCTTGTGGCC
Construction of the *bp3011*$_{C101G}$ mutant (downstream region)	F: GGCCACAAGACCTGG**G**GCACCTACCGAGTACTGCAG<br>R: CGCGTGGATCCGAATTCGAGTGCCCGAAGAGCTGAAGCGGC
RT-qPCR analysis of *bp3011* expression	F: CACGGCAGCTTCACCATTG<br>R: CTGCAGGACCCGGTAGGT
RT-qPCR analysis of *bp2871* expression	F: ACCCGACGAACTGCTGAC<br>R: AGCGCCACGATGCTGAAG
RT-qPCR analysis of *bp1363* expression	F: CTGGTGCAGCTGTTTGTG<br>R: GAATGCTTTCAATCGCGCC
RT-qPCR analysis of *bp2026* expression	F: AGTTCGGCGTCCTGTACTG<br>R: GGTGAAGAAGACCTGGGACT
RT-qPCR analysis of *bp2027* expression	F: CACACCATCACCTTCTGG<br>R: CTTGACCGTGTGGATGG
RT-qPCR analysis of *bp2028* expression	F: TGTTCAGGCCGTAGAAGG<br>R: CTGTGGCATGCTGTATGG
RT-qPCR analysis of *bp3432* expression	F: ACCAATATCATCGCCTGTCC<br>R: GTAGTCGAGATCGTCGAAGC
RT-qPCR analysis of *bp3433* expression	F: TGGGACGGAGAATTGCG<br>R: AGGAAGCTGTCGAAACCC
RT-qPCR analysis of *ptxA* expression	F: CCAGAACGGATTCACGGC<br>R: CTGCTGCTGGTGGAGACGA
RT-qPCR analysis of *dsbA* expression	F: ATACCGAATCGAACTTGGC<br>R: GCATCCCAAGGTCTTTACC

RT-qPCR analysis of *dsbC* expression	F: TCACGGTCTACACCTTCC<br>R: GCACCATCCAGTCTTTCC

*F, forward primer; R, reverse primer; sequences shown in italics indicate restriction enzyme recognition sites added for cloning purposes; GGC codons for glycine are underlined, the point mutation (changing the TGC codon for cysteine) is in bold.
